# A preliminary analysis of the effect of the new rural cooperative medical scheme on inpatient care at a county hospital

**DOI:** 10.1186/1472-6963-13-519

**Published:** 2013-12-17

**Authors:** Chiyu Ye, Shengnan Duan, Yuan Wu, Huimei Hu, Xiaofang Liu, Hua You, Linghao Wang, Lennart Bogg, Hengjin Dong

**Affiliations:** 1 Center for Health Policy Studies, School of Public Health, Zhejiang University School of Medicine, Hangzhou 310058, China; 2 Department of Health, Care and Social Welfare, Malardalen University, Västerås, Sweden; 3 Global Health, Department of Public Health Sciences, Karolinska Institute, Solna, Sweden

**Keywords:** Health insurance, NCMS, Inpatient, Hospital, Universal health coverage, China, Equity, Length of hospital stay, Cost, Access, Utilization

## Abstract

**Background:**

China in 2009 committed to reach universal health coverage by promoting three forms of health insurance; NCMS for the rural population, UEBMI for formally employed urban residents and URBMI for other urban residents. NCMS has expanded to near universal coverage in rural China since launching in 2003. The objective of this study aimed to assess the effect of NCMS on inpatient care utilization from 2003 to 2012 at Longyou county hospital, Zhejiang province.

**Methods:**

The research was conducted at Longyou county, Zhejiang province. All registered inpatient admissions from January 1, 2003, to June 30, 2012, were included in the study. The PLSQL Developer software was used to select the interesting variables in the hospital information database and saved in an Excel 2003 file. The interesting variables included the patients’ general information (name, gender, age, payment method), discharge diagnosis, length of hospital stay, and expenditure (total expenditure and out-of-pocket payment). Two common diseases (coronary arteriosclerotic disease and pneumonia) were selected as tracer conditions.

**Results:**

292,400 rural residents were enrolled in the Longyou county NCMS by 2011, 95.4% of the eligible population. A total of 145,744 inpatient admissions were registered from 1 January 2003 to 30 June 2012. The proportion of inpatients covered by NCMS increased from 30.3% in 2004 to 54.2% in 2012 while the proportion of inpatients covered by UEBMI increased from 7.7% in 2003 to 14.7% in 2012. The average expenditure for UEBMI insured inpatients was higher than the average for NCMS insured inpatients, although the gap was narrowing. The average length of hospital stay increased every year for all inpatients, but was higher for UEBMI inpatients than for NCMS insured inpatients. For both tracer conditions the results were similar to the above findings.

**Conclusions:**

NCMS has improved coverage height for its enrollees and resulted in increased cost of care per inpatient admission at the county hospital. However, wide differences persist between the two insurance systems in coverage height. Both systems are associated with increasing lengths of stay and rising cost per inpatient admission. We found that around 30% of inpatients were not covered by any of the two public health insurance systems, which calls for further studies.

## Background

The medical and healthcare system is one of the most important parts of the social security system and it can be seen as an indicator of the level of a country’s social progress and development [1]. The 6th national population census in China showed that by 2010 the total rural population had reached 674 million, constituting 50.3% of the country’s total population of 1.339 billion. The huge differences in health and in access to care between urban and rural residents, which widened in the early health reform period, have been reported in several studies^a^. Clearly, it is of great importance to improve the access and affordability of medical and healthcare service for over 600 million rural residents [2]. In the late 1990’s, the Chinese Government began to explore how the rural cooperative medical scheme (CMS), which disintegrated with the People’s Communes in the market reforms starting around 1978, could be revived. China has since set up a social medical insurance system, consisting of three separate systems with their own systems of enrollment (coverage breadth), funding, definitions of benefits (coverage depth) and financial coverage (coverage height), including the urban employee-based basic medical insurance (UEBMI), the urban resident-based medical insurance (URBMI) and the new cooperative medical scheme (NCMS) for rural residents. The three systems differ in levels of premium collection, reimbursement levels and ceilings as well as in what services and which drugs are covered. The UEBMI typically covers both inpatient and outpatient expenditures, while both the URBMI and the NCMS were designed primarily to protect against inpatient fees. The Central Committee of the Communist Party of China and the State Council issued a Decision on further strengthening rural health care in national conference on rural activities in October, 2002. The Decision explicitly pointed out that the NCMS should be established in a phased manner. Governments at all levels should actively organize and guide the up-build of NCMS based on a comprehensive arrangement to address serious diseases, and paying attention to tackling poverty and new poverty caused by major communicable, endemic and other health issues [3]. Thus, the NCMS had a focus on inpatient services and high-cost medical events from the conception.

Nevertheless, 70% of the NCMS systems in China cover both inpatient and outpatient expenditures^b^. Due to inconsistent regional development, the social medical insurance system differs from one county to the other. Some counties have adopted a unitary system where all urban and rural residents are enrolled in an urban–rural residents’ insurance scheme and enjoy the same benefits. Other counties operate binary systems, where urban formal sector employees take part in the UEBMI, while non-employed urban residents and rural residents enroll in an urban–rural residents’ insurance scheme. Most regions, however, have set up all three systems [4,5]. In 2009 the new round of health reforms in China confirmed political and financial support to move decisively towards universal and equitable health coverage^c^.

Longyou County is located in the west of Zhejiang Province, in prosperous East China. In 2010, the county’s permanent population reached 362,400, among which 64.2% were rural residents [6]. In 2011, the per capita disposable income of urban residents and the per capita net income of rural residents were 22,270 Yuan (￥) and 10,149 Yuan respectively. The per capita GNP was 39,102 Yuan, ranking 54th among 79 cities and counties in Zhejiang Province [7].

The medical insurance system in Longyou county is a binary system, where urban formal sector employees register in the UEBMI, other urban residents and rural residents enroll in an urban–rural cooperative medical insurance, which is essentially a form of NCMS. In Zhejiang Province, the costs of UEBMI are shared by employers and employees. 10% of the total amount of salaries has to be paid to UEBMI monthly. Employers contribute 8% of salaries (including pensions for retired employees), employees contribute the remaining 2%. Deductibles are intended to reduce the risk of moral hazard (excessive demand from patients who are insensitive to costs). The deductible amount varies according to the level of hospital; 2000 Yuan for tertiary hospitals, 1500 Yuan for secondary hospitals and 1000 Yuan for primary hospitals. At all hospitals, insurance covers 80% of the medical expenditures (85% for retired) above the deductible up to 20,000 Yuan; 85% (90% for retired) from 20,000 to 30,000 Yuan; and 90% (95% for retired) from 30,000 to the cap at 40,000 Yuan [8].

Longyou County was among the first counties in Zhejiang Province to pilot NCMS in 2003. The County Health Bureau put forward an experimental scheme in June 2003 and started implementation in February 2004. In 2006 the local government reformed the scheme. The revised scheme expanded the coverage of the insurance (coverage depth and height) and the eligibility of beneficiaries (coverage breadth). A family as a unit, urban and rural residents, whose households were registered (hukou) at Longyou could join the revised scheme. Further, the individual premiums and the deductibles were reduced, incentives for continuous insurance coverage were introduced, and adjustments were made to the cap and to subsidies. The government adjusted the NCMS policy again in 2008, raising the deductible for outpatient care and providing two voluntary schemes: basic insurance and major diseases insurance. In 2011, in response to the guidelines issued by Zhejiang Provincial Health Bureau, Longyou government revised the NCMS policy again. The new revised policy increased the local government’s subsidies substantially, reduced the deductibles for outpatient and for inpatient care, and integrated the basic insurance and major diseases insurance schemes (Table 1, Table 2).

**Table 1:** NCMS schemes of Longyou County 2004-2012

**Insurance scheme**	**Funding resources**	**2004-05**	**2006-07**	**2008**	**2009**	**2010**	**2011**	**2012**
**Overall scheme**	**Government (￥per capita)**	30	40				188	260
	**Individual (￥per capita)**	30	20				80	120
**Basic scheme**	**Government (￥per capita)**			80	94	120		
	**Individual (￥per capita)**			40	40	40		
**Major disease scheme**	**Government (￥per capita)**			80	100	120		
	**Individual (￥per capita)**			160	160	160		

**Table 2:** Longyou County NCMS reimbursement policies for inpatient care in 2004-2012

		**2004-05**	**2006-07**	**2008**	**2009**	**2010**	**2011**	**2012**
**Deductible (￥)**								
	**Township hospital**	1000	500	500	500	1000	300	300
	**Special hospital**	1000	500	500	500	1000	800	600
	**County Hospital**	1000	500	500	500	1000	1000	1000
**Reimbursement rate (%)**								
**Overall scheme**	**Deductible-￥2000**	15	15				60	70
	**￥2001-5000**	25	25				60	70
	**￥5001-10000**	35	35				60	70
	**￥10001-25000**	45	45				60	70
	**￥25001-**	60	60				60	70
**Basic scheme**	**Deductible-￥2000**			30	30	30		
	**￥2001-5000**			55	55	30		
	**￥5001-10000**			55	55	55		
	**￥10001-**			55	55	55		
**Major disease scheme**	**Deductible-￥2000**			35		35		
	**￥2001-5000**			60	60	35		
	**￥5001-10000**			60	60	55		
	**￥10001-**			80	85	85		
**Cap (￥10,000)**								
	**Overall scheme**	2	2				10	15
	**Basic scheme**			3	4	5		
	**Major diseases scheme**			6	8	10		

In order to understand the effect of NCMS on access and utilization of inpatient care, we collected data for all inpatient admissions at Longyou County Hospital from 2003 to 2012. The data was analyzed to assess the policies’ influence on rural inpatients’ utilization of medical services with an objective to provide information for policy development and decision-making. Our assumption was that after the implementation of NCMS, the access and utilization of inpatient services at the County hospital would increase gradually and the gap in the access and utilization of services between NCMS and UEBMI would narrow gradually.

## Methods

### Study site

The research was conducted at Longyou County, Zhejiang Province. The county was chosen because of its convenient geographic location in Zhejiang Province and because it was one of the pilot counties to implement NCMS. The Longyou County Hospital was chosen because it is the leading hospital in the county in terms of number of inpatient admissions and the scope of services and because it was one of the first hospitals contracted by NCMS. We only chose one county hospital because county hospitals provide the best health care services with the highest quality, compared to township hospitals and village health stations in rural China. We did not include township hospitals or other health care facilities because our purpose was to use a typical example to study the effects of NCMS on the use of the best health care services with the highest quality in rural area.

### Data collection

All registry inpatient admissions, totaling 145,744, from January 1, 2003, to June 30, 2012, were included in the study. The PLSQL Developer software was used to select the interesting variables in the hospital information database and saved in an Excel 2003 file. The interesting variables included patient’s general information (name, gender, age, payment method), discharge diagnosis, length of hospital stay, and expenditure (total expenditure and out-of-pocket payment).

Two diseases among the leading ten diagnoses were further selected for diagnosis-related analysis in order to control for the confounding factor of diseases pattern. The two selected diseases were coronary arteriosclerotic disease and pneumonia. A document study was made to collect information about policies and enrollment of the Longyou County NCMS from 2004 to 2012.

### Data analysis and indicators

The enrollment rate of NCMS, the composition of NCMS inpatients in the hospital, length of hospital stay, total hospital expenditure, out-of-pocket expenditure and the percentage of medical insurance reimbursement were analyzed to reflect the influence of NCMS on inpatient care utilization.

We compared the differences in the indicators between NCMS and UEBMI, because they are the main types of healthcare financing schemes in the county, but also in the whole of China. We grouped the remaining patients into ‘Other’ which includes those covered by private health insurance scheme and uninsured who pay user fees by ‘out-of-pocket’; however, we did not analyze the ‘Other’ group as it was a heterogeneous group.

This study has been approved by ethical committee of Zhejiang University School of Public Health. The ethical approved number is YG 2012007.

## Results

### NCMS enrollment

**Figure 1:**
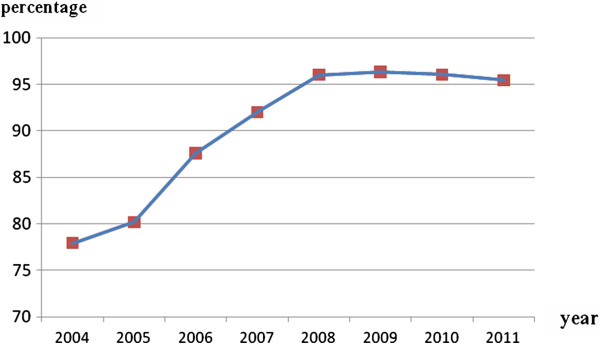
The NCMS enrollment rate at Longyou county from year 2004 to 2011.

The total number of NCMS enrollees at Longyou county was 293,392 in 2011 [9]. In other words, 95.4% of the eligible population were enrolled, an increase by 17.5% over that of 2004 (77.9%) [10] (Figure 1). We also found that there was a little reduction of the enrollment rate after 2009, which may be due to the development of private health insurance at Longyou, resulting in some drop-outs of NCMS membership.

### Composition of inpatient

The number of inpatients at the county hospital increased year by year since 2003, as the NCMS population coverage increased. The percentage of NCMS inpatients rose from 30.32% in 2004 to 54.18% in 2012, with an annual average growth rate of 7.5% (Table 3).

**Table 3:** Inpatients covered by different schemes from 2003 to 2012 at Longyou county hospital

**Year**	**Number of inpatients**	**NCMS (%)**	**UEBMI (%)**
**2003**	8075	/	621(7.69)
**2004**	9130	2768(30.32)	951(10.42)
**2005**	11373	3577(31.45)	1414(12.43)
**2006**	11771	4012(34.08)	1479(12.56)
**2007**	13557	4758(35.10)	1611(11.88)
**2008**	15945	5707(35.79)	1954(12.25)
**2009**	19560	7980(40.80)	2314(11.83)
**2010**	21581	9067(42.01)	2695(12.49)
**2011**	22330	10635(47.63)	3132(14.03)
**2012 1**^**st **^**half**	12422	6730(54.18)	1820(14.65)

### Hospital expenditure

**Figure 2:**
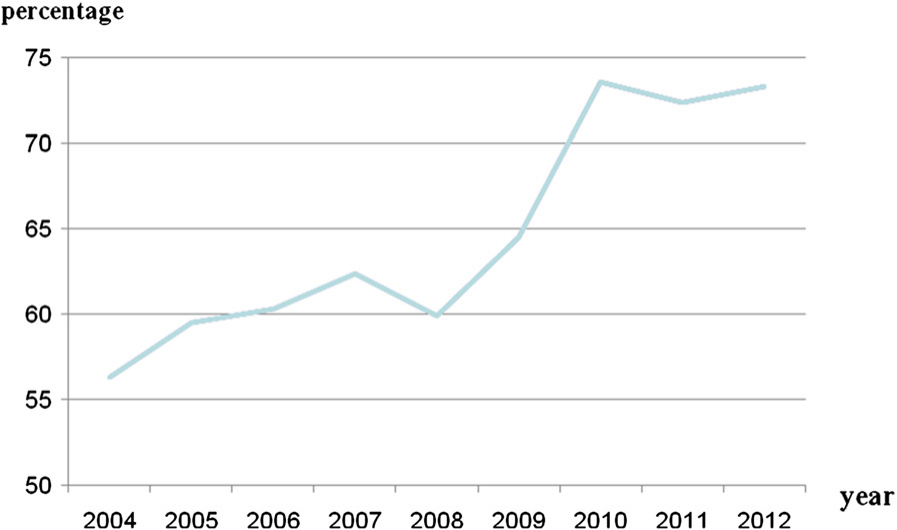
Average inpatients’ expenditure per admission of NCMS patients as a proportion of that of UEBMI patients.

Hospital expenditure at Longyou county hospital increased yearly and the gap in expenditure between NCMS insured inpatients and UEBMI insured inpatients decreased. For NCMS insured inpatients, the average expenditure per admission in 2004 was 4,389 yuan and in 2012 it was 6,853yuan, representing an annual increase of 5.7%; for UEBMI insured inpatients, the average expenditure per admission was 7,799 yuan in 2004 and 8,456 Yuan in 2012, an annual increase of 1.1%. NCMS insured inpatients’ hospital was 56.3% of the UEBMI insured inpatients’ in 2004, increasing to 73.3% in 2012 (Figure 2).

The average rate of reimbursement improved yearly both for NCMS inpatients and for UEBMI inpatients. The improvement was more pronounced for NCMS inpatients, from an average reimbursement rate of only 12.88% in 2004 improving to an average of 51.27% in 2012. It was still lower than that of UEBMI insured inpatients (up from 51.71% in 2003 to 76.05% in 2012), but the gap was narrowing (Table 4).

**Table 4:** Average inpatients’ expenditure from 2003 to 2012 at Longyou county hospital

**Year**	**NCMS**			**UEBMI**		
	**Expenditures (￥)**	**Out-of-pocket payment (%)**	**LOS (days)**	**Expenditures (￥)**	**Out-of-pocket payment (%)**	**LOS (days)**
**2003**	/	/	/	7084.56	3421.31(48.29)	10.84
**2004**	4389.21	3823.72(87.12)	8.90	7798.53	3604.01(46.21)	10.76
**2005**	4607.92	3974.15(86.25)	9.13	7740.90	3483.90(45.01)	10.91
**2006**	4704.75	3991.66(84.84)	9.17	7804.72	3326.29(42.62)	11.29
**2007**	5071.22	4180.85(82.44)	9.33	8132.51	3153.51(38.72)	12.03
**2008**	4942.99	3975.69(80.43)	10.32	8295.39	2909.24(35.07)	11.95
**2009**	5434.42	4121.90(75.85)	10.05	8423.06	2925.79(34.74)	11.76
**2010**	6150.28	4073.60(66.23)	10.21	8361.80	2395.89(28.65)	11.89
**2011**	6247.31	3537.29(56.62)	10.43	8632.75	2304.51(26.69)	12.06
**2012 1**^**st **^**half**	6195.72	3019.27(48.73)	10.78	8456.21	2025.42(23.95)	11.95

The average expenditure for pneumonia inpatients covered by NCMS increased every year in general although there were some variations; similarly, the average expenditure for pneumonia inpatients covered by UEBMI increased in general also although they decreased after year 2010 and the highest expenditure was in 2007. The reasons for these irregular variations are not clear. Furthermore, these irregular variations in the patients covered by UEBMI makes the gap in the expenditure between the two systems changed irregularly. The percentage of expenditure per inpatient covered by NCMS over that covered by UEBMI was 45.15% in 2007 (the lowest) to 101.25% in 2012 (the highest) (Table 5).

**Table 5:** Average inpatient expenditure and length of hospital stay for pneumonia patients at Longyou county hospital

	**UEBMI**				**NCMS**			
	**Expenditure (￥)**	**Out-of-pocket payment (%)**	**Hospital stay (day)**	**Expenditure per day (￥)**	**Expenditure (￥)**	**Out-of-pocket payment (%)**	**Hospital stay (day)**	**Expenditure per day (￥)**
**2004**	4786.32	2147.93(44.88)	10.36	462.00	4208.13	3786.05(89.97)	8.65	486.49
**2005**	4956.01	2113.56(42.65	10.45	474.26	4430.15	3889.23(87.79)	8.14	544.24
**2006**	4869.11	2032.97(41.75)	11.08	439.45	4275.56	3653.04(85.44)	7.46	573.13
**2007**	9935.18	2990.43(30.10)	14.67	677.24	4485.92	3803.16(84.78)	9.03	496.78
**2008**	6908.14	2561.68(37.08)	13.11	526.94	4362.49	3693.41(84.66)	8.20	532.01
**2009**	7080.39	2416.01(34.12)	14.02	505.02	5596.96	4375.81(78.18)	10.28	544.45
**2010**	7299.08	2277.33(31.20)	13.87	526.25	5875.66	3874.93(65.95)	10.39	565.51
**2011**	6458.69	1669.90(25.86)	12.68	509.36	5590.74	3099.93(55.45)	9.84	568.16
**2012 1**^**st**^**half**	5830.09	1427.57(24.49)	10.17	573.26	5903.05	2596.72(43.99)	10.38	568.69

The average inpatient expenditure for the NCMS insured patients who suffered from coronary arteriosclerotic disease continually increased from 4165.88 Yuan in 2004 to 5763.27 Yuan in 2012, but the expenditure was still lower than that of UEBMI inpatients (Table 6). The gap between the two insurance schemes, however, was narrowing, from 30.29% (1–69.71%) in 2004 to 22.67% (1–77.33%) in 2012. It is also found that the expenditure for inpatients covered by UEBMI in 2007 was the highest also, the reason so far is not clear.

**Table 6:** Average inpatient expenditures and length of stay for coronary arteriosclerotic disease patients at Longyou county hospital

	**UEBMI**				**NCMS**			
	**Expenditures (￥)**	**Out-of-pocket payment (%)**	**Hospital stay (day)**	**Expenditures per day (￥)**	**Expenditures (￥)**	**Out-of-pocket payment (%)**	**Hospital stay (day)**	**Expenditures per day (￥)**
**2004**	5975.63	2437.56(41.79)	11.74	509.00	4165.88	3700.05(88.82)	8.16	510.52
**2005**	6432.74	2455.68(38.17)	12.35	520.87	4295.42	3696.74(86.06)	8.32	516.28
**2006**	5395.55	1979.51(36.69)	11.51	468.77	4319.35	3685.26(85.32)	7.97	541.95
**2007**	7547.08	2497.08(33.09)	14.83	508.91	4431.03	3712.51(83.78)	8.10	547.04
**2008**	6773.92	2172.47(32.07)	13.76	492.29	4303.31	3515.45(81.69)	8.00	537.91
**2009**	6907.57	2204.57(31.92)	13.17	524.49	4813.26	3812.33(79.20)	8.70	553.25
**2010**	6160.60	1714.16(27.82)	11.16	552.03	4588.02	3130.85(68.24)	8.64	531.02
**2011**	5719.95	1307.83(22.86)	11.33	504.85	4974.80	2820.57(56.70)	8.80	565.32
**2012 1**^**st**^**half**	7452.66	1565.10(21.00)	13.00	573.28	5763.27	2611.24(45.31)	9.52	605.39

### Length of hospital stay

For NCMS insured inpatients, the average length of stay was 8.9 days in 2004 and 10.8 days in 2012, an annual increase by 2.4%. For UEBMI inpatients, the average length of stay was 10.8 days in 2004 and 12.0 days in 2012, an annual increase by 1.2% (Table 4). However, for the average expenditure per day, there was no significant difference between the two schemes. But for both diseases, we find the length of hospital stay for patients covered by UEBMI were the highest in 2007 during the period of 2004–2012 (Table 5, Table 6). This may be one reason that can explain why the expenditure for patients covered by UEBMI was extremely high in 2007 (Table 5, Table 6).

### ‘Out-of-pocket’ payment

For NCMS insured inpatients, the expenditures paid by ‘out-of-pocket’ decreased every year, from 88.82% in 2004 to 45.31% in 2012. However, it was still higher than that of UEBMI insured inpatients (Table 4). For UEBMI inpatients, the expenditures paid by ‘out-of-pocket’ also decreased every year, from 48.29% in 2004 to 23.95% in 2012 (Table 4). Take pneumonia inpatients as example, for NCMS insured inpatients, the expenditures paid by ‘out-of-pocket’ decreased from 89.97% in 2004 to 43.99% in 2012, while UEBMI insured inpatients decreased from 44.88% in 2004 to 24.49% in 2012 (Table 5). Inpatients who suffered from coronary arteriosclerotic disease complied with the same rules, ‘out-of-pocket’ payment decreased from 88.82% to 45.31% for the insured by NCMS, and from 41.79% to 21.00% for the insured by UEBMI (Table 6).

## Discussion

The main findings of this study are that from year 2003 to 2012 the enrollment rate of NCMS for the eligible population increased, the proportion of NCMS covered inpatients at the county hospital increased also, the reimbursed proportion of the medical expenditures increased also, leading to a reduction in ‘out-of-pocket’ payment and a narrowing of the gap in the utilization of inpatient services between NCMS insured and UEBMI insured. It is clear that access to health care has improved and the risk of financial catastrophe may have been reduced as a consequence of the reduction in ‘out-of-pocket’ expenditure.

The County hospital offers the best medical services at Longyou county. For healthcare seekers, it is essential that the cost of care is affordable [11]. For rural residents at Longyou county, the NCMS system has made the cost affordable although the inequity in the access to health care between different healthcare financing schemes still exists. The NCMS insured, in general, pays, half of the cost of inpatient care, while UEBMI insured only pay one fifth of the cost. The good thing is that, however, with the increasing reimbursement rate for NCMS insured, more patients will seek care at county hospital, obtaining better medical care [12,13].

Our study supports the findings from earlier studies [2]. They find that the NCMS reform may alleviate rural patients’ economic burden caused by diseases and improve their utilization of medical services. It is well known that the cost of medical care is the leading cause of poverty in China, as in many other countries. Inpatient care is the most costly component of medical services and long hospital stays can lead to catastrophic costs. For this reason universal pooling of resources in health is of vital importance both to reduce the risk of impoverishment and of barriers to care. At the early stage of the NCMS implementation, the percentage of government reimbursement was relatively low, below 20%, which implied that the protection from financial catastrophe was not sufficient and financial barriers were not removed. However, after the reform in 2008, which introduced the general insurance and the medical insurance for major diseases, the reimbursement percentage increased substantially. From 2008 to 2010, Longyou county government revised the NCMS policy every year to raise the percentage and decrease the gap of reimbursements. In 2011, the county government integrated the two insurance schemes (NCMS and URBMI, but the integrated one is still called NCMS) and canceled the piecewise reimbursement mode, benefitting patients who have a low cost by a higher reimbursement percentage. As a result, NCMS patients are now reimbursed on average by more than half of their hospital expenditure. With the increasing reimbursement proportions and yearly reductions of the ‘out-of-pocket’ payments, the disparity between rural and urban inpatients in terms of length of hospital stay and total hospital expenditure continued to decrease yearly.

In the case of pneumonia inpatients, 75.51% of the hospital expenditures were reimbursed for UEBMI patients in 2012, compared to 56.01% for NCMS pneumonia patients, yet in 2004, the gap was much larger. The trends are the same as the case of coronary arteriosclerotic disease. This further shows the effects of NCMS on reducing the financial gap between the two health care financing schemes.

Universal health coverage in China, the same as in other countries, includes population coverage (coverage breadth), services (benefits) coverage (coverage depth) and financial coverage (coverage height). Longyou county is making progress to meet the criteria for universal health coverage, especially in terms of coverage breadth; but still has a long way to go to reach other two types of coverage, especially in the coverage height. In this study, we only study the population coverage and financial coverage, but did not examine the services coverage. So, in this study, we cannot provide what is the difference in the use of medical services between the two schemes.

NCMS is one of the most important policies on universal coverage of health care in China [14]. The success of universal coverage, however, depends on how effective its design and implementation arrangements are in reaching population and affecting households’ health seeking behavior and abilities to take up benefits of universal coverage [15,16]. In 2011, NCMS has covered 95% of eligible population. However, the benefits package is still not comprehensive and the ‘out-of-pocket’ payment is still high. China still has a long way to reach a real universal coverage.

This is a typical study only using inpatient data from one county hospital. The results cannot be generalized from the methodology point of view. It is better to include more hospitals. However, the results are quite interesting and show the trends of improving equity in the utilization of health care from different health care financing schemes.

## Conclusions

The coverage rate of NCMS (coverage breadth) for the eligible population has increased sharply after the implementation of the scheme. The proportion of admitted inpatients at the county hospital who are insured by NCMS has increased and the proportion of inpatient medical expenditures covered by NCMS has increased resulting in a decreased gap in ‘out-of-pocket’ expenditures between NCMS and UEBMI inpatients. NCMS is clearly of great importance to reduce inequity in access to health care with the ultimate goal to reach universal health coverage, nevertheless, more needs to be done to equalize the two systems. The benefits packages need to be further studied to explore differences in coverage depth and efforts are required to reduce the differences in coverage height. The urban employees’ health insurance system (UEBMI) covers more than 75% of inpatient care costs, while the system for non-employed (NCMS) covers only less than half the costs. Both systems are associated with increasing lengths of stay and increasing cost per inpatient admission, which should cause some concern. We noted that although the theoretical enrollment in health insurance is 95.4% of the eligible population, hospital records show that around 30% of the admitted inpatients were not covered by any of the two public systems.

## Endnotes

^a^Bogg L, Dong HJ, Wang KL, Cai WW and Diwan V, The Cost of Coverage: Rural Health Insurance in China, 1996, Health Policy and Planning, 11(3): 238–252.

^b^Meng Qingyue and Tang Shenglan, Universal coverage of Health Care in China: Challenges and Opportunities, 2010, World Health Report (2010) Background Paper 7, WHO, Geneva.

^c^CCPC and the State Council (The Chinese Communist Party Committee and the State Council). Guidelines for Deepening Health Systems Reform. Beijing, 2009-03-17.

## Competing interests

The authors declare that they have no competing interests.

## Authors’ contributions

CY and HD designed and implemented the study and did the first draft of the manuscript. CY, HD, SD, YW, WH, HY, XL and LW participated in the data collection and the statistical analysis. CY, HD, SD, YW, WH, HY, XL and LW have read and approved the final manuscript. LB has read the first draft and did a substantial revision for the final submission. All authors read and approved the final manuscript.

## Pre-publication history

The pre-publication history for this paper can be accessed here:

http://www.biomedcentral.com/1472-6963/13/519/prepub
